# Women's liberation and the rhetoric of "choice" in infant feeding debates

**DOI:** 10.1186/1746-4358-3-10

**Published:** 2008-08-04

**Authors:** Bernice L Hausman

**Affiliations:** 1 Department of English, College of Liberal Arts and Human Sciences, Virginia Tech, Blacksburg, Virginia, USA

## Abstract

This short essay examines infant formula marketing and information sources for their representation of "choice" in the infant feeding context, and finds that while providing information about breast and bottle feeding, infant formula manufacturers focus on mothers' feelings and intuition rather than knowledge in making decisions. In addition, the essay considers how "choice" operates in the history of reproductive rights, shifting the discourse from a rights-based set of arguments to one based on a consumerist mentality. Utilizing the work of historian Rickie Solinger and a 2007 paper for the National Bureau of Labor Statistics, I argue that the structure of market work, and not abstract maternal decision making, determine mothers' choices and practices concerning infant feeding. For true freedoms for mothers to be achieved, freedoms that would include greater social provisions for mothers, our culture will have to confront how structural constraints make breastfeeding difficult, as well as how the concept of choice divides mothers into those who make good choices and those who do not.

## Debate

I began to prepare this presentation with the idea that choice, as a concept, has been appropriated by infant formula companies in connection with women's liberation. Yet websites by Ross Laboratories, Mead Johnson, and Nestle/Carnation present "the choice to feed a baby with infant formula" as being increasingly under fire, presumably by strong medical evidence about the health contributions of breastfeeding. In this context, "choice" is presented as based on maternal feelings; mothers make good choices when they follow their sensibility, or "heart," rather than their heads.

Specifically, current rhetoric from infant formula company websites articulates choice defensively, or with qualifiers about what is actually important in infant feeding. For example, in some articulations, love is more significant than breast milk or infant formula, which displaces the choice altogether onto the question of maternal emotions and, at some level, fitness. Implicitly, there's the sense of attack – if you feed with formula, you do not love your baby enough. The response, then, is to argue that it is love that counts in infant feeding and care, not what goes into the baby. Palpable in this rhetoric is the sense that mothers feel burdened by the need to demonstrate that they are good mothers by the fact that they love their children.

Important in these materials is the notion of an "informed decision" to breastfeed or feed with infant formula. The information on each site about advantages and disadvantages of each choice exists to help mothers make these "informed decisions." However, the notion of an informed decision is undermined by the idea that decisions about infant feeding are made by the heart, not the head: in the end, information is not really the deciding factor. For example, on Mead Johnson's Enfamil website, after the discussion of "Choosing to Breastfeed," the page ends with the following statement: "Now that you're familiar with some of the advantages of breastfeeding, you have the added advantage of making an informed decision. In the end, of course, you'll do what your heart tells you. You can't go wrong. After all, that's the organ that's pumping out all that love for your baby" [1].

Another Enfamil web page discusses "It's Your Family's Decision": "Only mom and dad know what will work best for their family. So, be confident in the choice that you make. The best way to deal with people who question your choice is to simply tell them, politely but firmly, that you have discussed how to feed your baby with your baby's doctor. Feel good about your decision and be confident your baby is getting the essential nutrients he needs" [2]. Reading this I imagine what discussions of abortion would be like in the same register: "Look, I've discussed my decision to terminate this pregnancy with my doctor and she agrees it's a good idea, healthy for me. It's the right decision for my family as well. I'm confident in this decision, so you need to butt out." To anyone familiar with the abortion debates, it's clear that infant formula makers champion a rhetoric around "choice" that used to be a common approach to abortion rights but which is difficult to promote publicly today.

Thus "choice" concerning infant feeding in these product-oriented informational sites echoes some aspects of the discourses of reproductive rights struggles. Some feminist historians have struggled with the concept of choice with respect to abortion rights, seeing it as a figuration of consumerism right from the 1970s, when *Roe v. Wade *made abortion legal in the USA. Rickie Solinger has shown that "choice" (as a concept) obscures the importance of rights with respect to abortion and reproductive freedom. In an article "Poisonous Choice," and her subsequent book *Beggars and Choosers*, Solinger criticizes the use of "choice" to articulate abortion rights, and demonstrates how "choice" operates to stratify mothers into categories of good and bad choosers (i.e. good and bad mothers) [3,4]. First, she demonstrates how the concept of "choice" moved abortion rights from a rights framework to one focused on women as consumers, arguing that choice was already being used in terms of consumer privilege with respect to reproductive rights in the initial aftermath of *Roe v. Wade*. The use of "choice" instead of "abortion rights" made an alliance between the right to control one's fertility through pregnancy termination with the consumerist connotations of "choice." Any decision a woman makes about reproduction thus becomes vaguely connected to her "rights" as a consumer, rather than her rights as a human being [3].

Then Solinger argues that the articulation of abortion rights as "choice" opened up the possibility of women being criticized for making wrong choice*s*. The rhetorical links of "choice" to consumerism, and the ability to turn the discourse of choice into one of blame led to "choice" becoming a very narrow political slogan that foregrounded the experience of middle-class women and made all other women vulnerable, because what are construed as "rights" in this scenario turn out to be privileges available to very few [4].

So what are the links between infant formula use and women's liberation, then? Is there no connection between infant feeding choice and women's freedom? It seems to me that there does not need to be a *rhetorical *link made by infant formula companies between replacement feeding and maternal freedoms. The link exists materially in the structure of market work. In a recent "working paper" published in 2007 by the U.S. National Bureau of Labor Statistics, Albanesi and Olivetti demonstrate that life decisions and practices associated with increasing freedom for women – largely understood as participation in waged labor – were made possible by technological advances perceived to free women from domestic burdens, including the reproductive burden of breastfeeding [5]. The resurgence of breastfeeding since 1970 has occurred in the context of women's increased labor force participation, which means that breastfeeding is configured as a "choice" to be made against a structure – market work – that became available to women largely when they stopped breastfeeding. Since replacement feeding was believed to solve the problem of mothers' embodied responsibility to feed their babies, other kinds of solutions to mothers' market work were not structured into the economy. Thus, "infant feeding choice" is figured as a personal decision, which is why, as Albanesi and Olivetti remark, while the rate of breastfeeding initiation in 2004 "is comparable to those observed in the 1920s, the duration of breastfeeding is now much lower" [5].

Additionally, by identifying the development and marketing of infant formula as a necessary corollary to women's increased involvement in waged labor, Albanesi and Olivetti demonstrate how infant formula is linked to perceptions of women's liberation [5]. In the United States, liberation and freedom are connected with economic self-sufficiency. In breastfeeding advocacy we see how much economic self-sufficiency makes breastfeeding a difficult practice to sustain for most women [5]. This is why, in my view, the structure of market work is one thing that must change in order to accommodate true maternal freedom, which would involve a relatively unconstrained ability to breastfeed one's children.

What would real liberation for mothers entail? Structural changes to market work and greater social provisions, sponsored by the state, for mothers and their families, would lead to increased freedoms for mothers. However, even societies with generous maternity provisions continue to identify breastfeeding as a risky behavior, thus perpetuating a widespread distrust of nursing. This suggests that instituting breastfeeding as a norm will entail cultural changes in the way we think about women and their bodies – in public spaces, as nurturers, as sexual beings, as autonomous adults who are nevertheless physically connected to infants. Most perceived risks about women's bodies as breastfeeding mothers surround anxieties about modern maternity. Cultural change is needed, as feminism has always argued, to perceive women as responsible individuals fully capable of living their lives as free people. What we need to do is find new definitions of freedom that incorporate dependence, chance, and connectedness as essential and ordinary, so that women's rights as mothers are not abrogated by normative expectations of their fitness to carry out those roles.

Significantly, choice is not liberation. If Rickie Solinger is correct, "choice" has only meant the right of access to abortion for middle class women, and thus it has never really meant choice, nor rights, in a universal sense [3]. "Choice" in infant feeding method has not liberated women from the burdens of maternity, although many women have benefited from entry into waged labor made possible under current constraints by replacement feeding. It is possible that the pressures felt by women who do not breastfeed – who feel that others look at them as if they have made a "bad choice" – are a legacy of the way choice rhetorically operates in relation to motherhood, functioning to distinguish mothers who choose well from those who do not. Infant feeding rhetoric on the formula manufacturers' websites responds to this perceived stigma by suggesting that women choose well when they use their emotions. All choices, when made from the "heart," are good choices, especially when women make choices perceived to be poor from a medical point of view.

Constraints on breastfeeding are far harder to overcome for poor women, young women, women of color, and women with less education than for those women with education, resources, and more life experience. We can apply Solinger's analysis of abortion to infant feeding and notice that "choice" in infant feeding method operates to distinguish women who make "good choices" from those who do not, as if those choices are unconstrained. Infant feeding choices – whether made by "heart" or "head" – are practiced in the context of the social, cultural, and economic forces that structure most people's daily lives and intimate decisions. It is our responsibility, as feminists, to identify the constraints that reveal the "choice" itself to be not so much a choice but a class privilege, and then to figure out how to challenge the status quo that makes it so.

## Competing interests

The author declares that she has no competing interests.
